# A Correlation Analysis between Arch Width and Molar Inclination Using Cone-Beam Computed Tomography Transverse Measurements: A Cross-Sectional Study

**DOI:** 10.3390/diagnostics13111875

**Published:** 2023-05-27

**Authors:** Farraj Albalawi, Reem Alwakeel, Samah Alfuriji, Nasser D. Alqahtani, Rana M. Barakeh, Amjad AlGhaihab, Suliman Alsaeed

**Affiliations:** 1 Preventive Dental Science Department, College of Dentistry, King Saud bin Abdulaziz University for Health Sciences, Riyadh 11426, Saudi Arabia; furijis@ksau-hs.edu.sa (S.A.); suliman.as2@gmail.com (S.A.); 2 King Abdullah International Medical Research Center, Ministry of National Guard Health Affairs, Riyadh 11481, Saudi Arabia; reemleena@gmail.com (R.A.); amjaadalghaihab@gmail.com (A.A.); 3 Saudi Board of Orthodontics and Craniofacial Orthopedics Resident, National Guard Hospital (NGHA), Riyadh 11426, Saudi Arabia; 4 College of Dentistry, King Saud bin Abdulaziz University for Health Sciences, Riyadh 11426, Saudi Arabia; 5 Pediatric Dentistry and Orthodontic Department, College of Dentistry, King Saud University, Riyadh 11545, Saudi Arabia; nasserdm@ksu.edu.sa (N.D.A.); rana.barakah@gmail.com (R.M.B.); 6 Department of Maxillofacial Surgery and Diagnostic Sciences, College of Dentistry, King Saud bin Abdulaziz University for Health Sciences, Riyadh 14611, Saudi Arabia

**Keywords:** CBCT, dental compensations, imaging, orthodontics, posterior crossbite

## Abstract

A new era in 3-dimensional analysis has begun with the use of cone-beam computed tomography (CBCT) in orthodontics, which promises to provide a more thorough understanding of the craniofacial skeletal architecture. This study aimed to investigate the correlation between the transverse basal arches discrepancy and dental compensation by utilizing CBCT width analysis. An observational study was conducted to retrospectively review 88 CBCT scans of patients presented to dental clinics from 2014 to 2020 obtained from the Planmeca Romexis x-ray system at three centers. Dental compensation data across normal and narrow maxillae were analyzed and a Pearson correlation was used to find the relationship between molar inclination and width difference. Significant maxillary molar compensation differences were observed between the normal maxilla and narrow maxilla group, where the amount of dental compensation (164.73 ± 10.15) was higher in the narrow maxilla group. A significant negative correlation (r = −0.37) was observed between width difference and maxillary molar inclination. Maxillary molars were tipped buccally to compensate for the reduced maxillary arch width. These findings are important to determine the amount of needed maxillary expansion taking into account the buccal inclination while treating cases.

## 1. Introduction

Successful orthodontic treatment relies on accurate diagnosis of the craniofacial skeleton in all three planes of space—sagittal, vertical, and transverse. However, the transverse plane is often overlooked during diagnosis, even though it can play a significant role in determining the underlying cause of asymmetric or symmetric transverse problems [1,2,3]. Such problems may originate primarily from skeletal, dental, or functional factors, or a combination thereof. Causes of maxillary constriction can arise from genetic factors or certain habits, such as mouth breathing and thumb/digit sucking [4,5,6]. This could explain why females have a higher prevalence of posterior crossbites compared to males, due to the reported higher prevalence of thumb sucking among girls [7]. Additionally, a low tongue posture due to having a tongue tie can also contribute to this condition [6]. Research has shown that almost 25% of posterior crossbite cases are linked with the habit of thumb sucking, while dummy/pacifier sucking is even more closely associated with it [8]. If these habits persist for less than one year, there may not be any significant adverse effects on occlusion; however, if they continue for over four years, patients should expect a posterior crossbite and an anterior open bite to occur [9]. Another common cause of posterior crossbites is upper airway obstruction, due to large adenoids or tonsils, that results in mouth breathing and subsequently affects the equilibrium of forces around the maxilla that might result in restriction of the transverse growth of the upper jaw [6]. This transverse growth of the maxilla is of particular concern as it grows the least and stops growing the soonest [10,11,12].

A common transverse skeletal discrepancy is insufficient maxillary arch width, which can be detected clinically as a posterior crossbite. These crossbites can be either unilateral or bilateral and can be due to irregularities from the upper or lower jaws/arches or both. This complex malocclusion reflects the importance of careful evaluation of teeth articulation and dentition, in order to reach accurate diagnosis, and hence, an optimal treatment planning. The prevalence of posterior crossbites varies in the literature. It can be as low as 7% and as common as 23% [4,8,13]. The most common observation of posterior crossbites is a unilateral posterior crossbite with a mandibular functional shift [4,13,14]. However, most of these unilateral posterior crossbites are in fact bilateral but associated with a functional shift that results in the presentation of a unilateral crossbite [15]. This functional shift results from premature contact between the upper and lower teeth, which encourages the lower jaw to shift to either the right or left side to achieve maximum intercuspation [16]. These interferences usually arise from the primary canines [6]. However, functional shifts can also lead to forward positioning of the mandible, known as “pseudo-class III” [6]. During examination, clinicians should examine the dental midlines and chin position to evaluate for possible shifting of the lower jaw, which can result in facial asymmetry later on, if left untreated [17,18].

Although a narrow maxilla often results in posterior crossbites, there are certain situations where dental compensation can mask the underlying posterior crossbites [6,15]. This can be presented by the buccolingual axial inclinations of posterior teeth, leading to dental compensation [6,15]. Upper posterior teeth will tend to tip toward the buccal side, while lower posterior teeth will tend to tip toward the lingual side, in order to compensate for the discrepancy in the transverse plane [6,15]. A constricted maxillary arch is observed most frequently in Class III patients and least in Class I patients [19,20]. Unfortunately, transverse skeletal discrepancies have been largely ignored because radiographs used for diagnosis, such as panoramic and cephalometric radiographs, are mainly concerned with sagittal and vertical planes analysis [2,21].

One of the traditional techniques to assess the transverse skeletal relationship is by utilizing data gathered from posteroanterior cephalometric radiographs. However, this imaging technique provides a two-dimensional image that is prone to projection errors and image magnification [22,23,24].

Similarly, casts have been used for transverse dental analysis by McNamara [25] and Andrews [26], but they do not provide an accurate measure of dentoalveolar compensation and may mask underlying transverse deficiencies [19].

Currently, we find ourselves in a new age where cone-beam computed tomography (CBCT) technology enables us to view all dimensions in a comprehensive 3D manner [27]. This technology allows for accurate measurement of both transverse skeletal analysis and dental axial inclination without the need for additional records. Consequently, we can investigate the correlation between a constricted maxillary arch, the presence of dental axial compensation, and a posterior crossbite. In a study conducted by Grunheid T. et al. [28], digital models were fabricated from CBCT scans using advanced software algorithms, which eliminated the need for traditional impressions. Additionally, CBCT scans can be used for surgical purposes such as fabrications of splints for orthognathic surgeries or trauma cases [29]. The technology has also significantly improved airway analysis, enabling 3D and volumetric analysis as per the findings of Ghoneima A. et al. [30]. CBCT analysis is beneficial in predicting anomalies, diagnosing, analyzing arch morphology, providing prognosis, and developing treatment plans. As such, this study aims to investigate the correlation between the transverse basal arches discrepancy, dental compensation, and the presence of posterior crossbite through CBCT width analysis.

## 2. Materials and Methods

This was a cross-sectional study approved by King Abdullah International Medical Research Center (Institutional Review Board Approval # RC20/410/R). The study sample was obtained from three different dental centers (King Saud University, King Saud bin Abdulaziz University for Health Sciences, and National Guard Health Affairs). All CBCT scans of patients taken between 2014 and 2020 were retrospectively analyzed. All CBCT scans were taken with teeth in occlusion using the Planmeca x-ray system with the following standardized exposure parameters: 90 kilovoltage, 11 milliamperage, 12 s, 0.3 mm voxel size. Patients aged 15–60 years old in permanent dentition, along with erupted permanent mandibular and maxillary first molars, who had good-quality CBCT scans previously taken for the maxilla and mandible were included. Patients who had a previous history of orthodontic or orthognathic surgery treatment, were missing first permanent molars or premolars, had a craniofacial anomaly, had crowns or prosthesis on their upper or lower first molars, had implants, or had more than 5 mm upper or lower teeth crowding were excluded. A sample size calculation was performed and yielded 40 cases per group (narrow maxilla: 40, normal maxilla: 40) required for a significance level of 5% and 90% power to detect a mean difference of 4 and a standard deviation of 5.5. The CBCT scans were analyzed using Planmeca Romexis software (version 4.6.2.R, Helsinki, Finland). This software involved a complete set of tools for image viewing, enhancement, evaluation, measurement in all planes, drawing, and annotations. A standardized method to orient the CBCT for analysis was used with the midpalatal suture as the vertical reference and the occlusal plane as the horizontal reference. When these two lines (midpalatal suture and occlusal plane) were perpendicular (90 degrees) to each other, slicing of the scan started and measurements were taken. The cross-sectional slicing was set to 0.5 mm, until the best fit of the right and left first molars was achieved.

**Figure 1:**
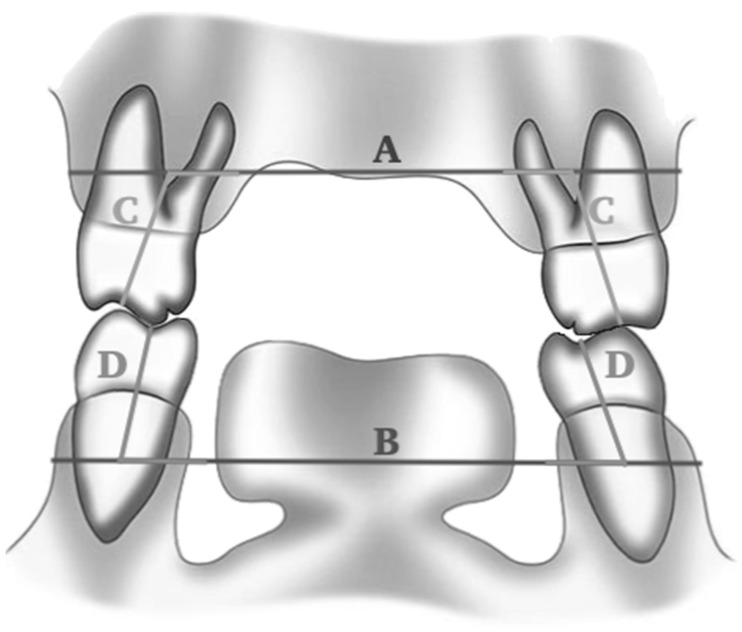
Maxillary width plane (**A**): measured by connecting the points on the buccal concavity cortexes of the maxilla at a vertical level halfway between the buccal alveolar crest and the buccal root apex of the maxillary first molar. Mandibular width plane (**B**): measured by connecting the points of the WALA ridge, the points on the buccal cortexes of the mandible at a vertical level halfway between the buccal alveolar crest and the apex of the mandibular first molar. Maxillary molar long axis (**C**): the line drawn between the deepest concavity between the buccal and palatal cusps and the furcation of the roots. Mandibular molar long axis (**D**): the line drawn between the deepest concavity between the buccal and lingual cusps and the root apex. Maxillary molar inclination was measured as the angle between maxillary width plane and maxillary molar long axis (**A**/**C**). Mandibular molar inclination was measured as the angle between mandibular width plane and mandibular molar long axis (**B**/**D**).

A modified measurement method from Tamburrino et al. [31] was used to measure the maxillary arch width, mandibular arch width, and buccolingual (B-L) axial inclination of maxillary and mandibular molars. A total of fifteen scans were randomly analyzed for repeated measurements. Two examiners assessed the measurements of the arch widths and molar inclinations. The intra-rater reliability was assessed by having each examiner perform a transverse CBCT analysis on each scan twice, at least a week apart. The inter-rater reliability was then determined by assessing the agreement between the two examiners. For the maxillary and mandibular arch width, measurements were taken by measuring the distance between first permanent molars on the WALA point at the basal bone level (Figure 1). Arch widths were analyzed by comparing the maxillary and mandibular arch widths and assessing the difference between them in mm, where the optimal maxillo-mandibular transverse difference is 5 mm in mature patients (14). To measure the teeth inclination, lines were drawn following the maxillary and mandibular molars’ long axis (middle of the tooth at the area of furcation) toward the maxillary and mandibular width plane, respectively. The molar axial inclination degree was the angle between the long axis of the first molar and the width plane (Figure 1). Furthermore, the dental compensation was measured as the sum of the inclinations of right and left molars of both the maxilla and mandible. The correlation between maxillary and mandibular width and B-L axial inclination of maxillary and mandibular molars was assessed to determine the dental compensation along with its relation to other dental findings. The transverse basal arches discrepancy was assessed as per Tamburrino et al. and hence, the study sample was divided into a normal maxilla group (maxillo-mandibular discrepancy = 0–5 mm) and a narrow maxilla group (maxillo-mandibular discrepancy ≤ 0 mm). The diagnosis of posterior crossbite was confirmed by examining the 3D skeleton model that was constructed by the software.

### Statistical Analysis

A standard descriptive statistic was calculated for each measurement with the SPSS program version 23, Chicago, USA. Normality of the data was assessed using the Shapiro–Wilk test. An independent t test was used to analyze the dental compensation of the maxilla and mandible across the two groups. A Pearson correlation was used to find the relationship between molar inclination and width difference. A Chi squared test was used to find out prevalence of posterior crossbite in normal maxilla and narrow maxilla groups and a *p* value of less than 0.05 was considered to be statistically significant. Cronbach’s alpha test was used to assess the inter-rater and intra-rater reliabilities of the measurements.

## 3. Results

**Figure 2:**
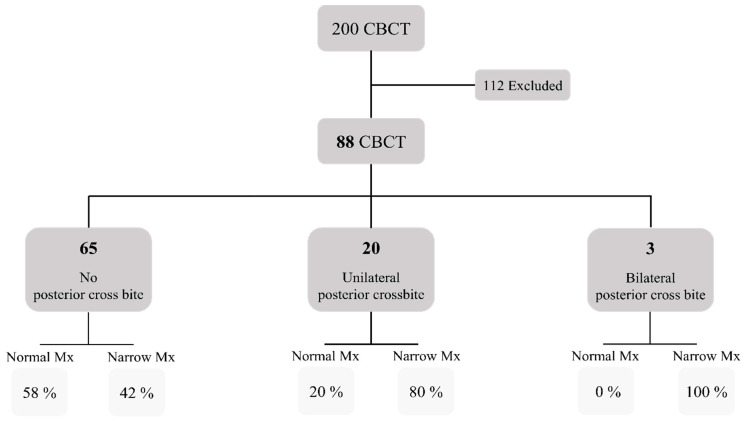
Flowchart for the distribution of the study sample.

A total of 200 CBCT scans were reviewed, of which 88 scans (44%) were eligible for analysis. There were 112 CBCT scans excluded (56%) due to either missing permanent upper or lower first molars, having implants in upper or lower molar regions, poor radiographic quality, or a history of orthodontic treatment. The mean age of the patients was 31.24 ± 13.97 years old; the majority were females (55%) as shown in Table 1. Cronbach’s alpha test showed that inter-rater and intra-rater agreement was highly reliable (α = 0.91) for the measurements chosen. Most of the cases (73.9%, 65 cases) had no posterior crossbite; 22.7% (20 cases) had unilateral crossbite, while 3.4% (3 cases) had bilateral crossbite. The demographics between the groups were different in terms of gender and age. The mean age of the bilateral crossbite group was significantly younger than either the unilateral crossbite group or the “no crossbite” group. In terms of maxillary and mandibular widths, the average width of the maxilla was similar for the “no crossbite” and “unilateral crossbite” groups with an average of 55 mm, but was narrower for the “bilateral crossbite” group with 51 mm. For the mandibular width, the width of the mandibular arch was around 55 mm for both the “no crossbite” and the “bilateral crossbite” groups, but wider for the “unilateral crossbite” group with a 58 mm average width. Overall, the difference between the maxillary and mandibular width was minimal for the “no crossbite” group (0.06 ± 3.39 mm) but was evident in the unilateral crossbite group with an average difference of 3.15 ± 3.94 mm, and even more pronounced in the bilateral crossbite group with an average difference of 4.25 ± 4.10 mm (Table 1). After analyzing the transverse basal arch discrepancy of all cases, 59% (52 cases) had a narrow maxilla, whereas the rest (41%, 36 cases) had a normal maxillary width as depicted in Figure 2 and Table 2.

**Figure 3:**
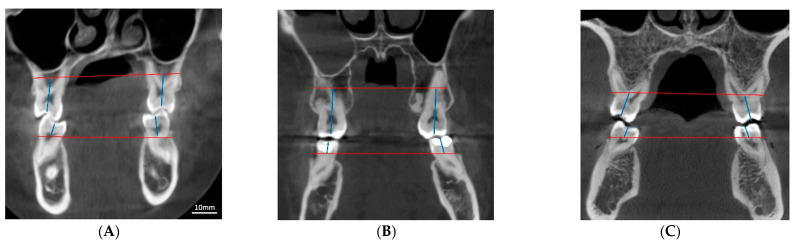
Example of different combinations of arch width and molar inclination. (**A**): Normal maxillary width and normal molar B-L axial inclination. (**B**): Constricted maxilla, normal molar B-L axial inclination, presence of bilateral posterior crossbite. (**C**): Constricted maxilla, buccally flared maxillary molars, absence of posterior crossbite. (The red lines in the CBCT images indicate the measured widths of the maxilla and mandible. The blue lines represent the inclinations of the molars on the CBCT images).

The maxillary molar compensation was statistically different between the normal and narrow maxilla groups. The mean maxillary molar compensation in the normal maxilla width group was observed to be 170.43 ± 10.51° while in the narrow maxilla group it was 164.73 ± 10.15° (*p*-value = 0.01) On the other hand, the mean mandibular molar compensation in the normal maxilla group was reported to be 204.54 ± 15.19° and in the narrow maxilla group it was found to be 208.64 ± 17.13° (*p*-value = 0.25), which is not statistically significant as depicted in Figure 3 and Table 3.

The correlation between width difference and maxillary molar inclination yielded a statistically significant negative correlation (r = −0.3), whereas a positive correlation (r = 117) was found between width difference and mandibular molar inclination without any statistical significance as depicted in Table 4.

## 4. Discussion

Malocclusion usually requires correction either in the sagittal, vertical, or transverse dimension. Since transverse facial growth is normally completed before vertical and sagittal growth, it is important to detect transverse discrepancies and correct any deficiency in early stages of growth [1]. Routine pre-treatment investigation and detection of transverse discrepancies is difficult since posteroanterior cephalogram (PA ceph) is rarely used in orthodontic practice compared to lateral cephalogram. PA ceph analysis is used to evaluate breadth, morphology, symmetry, shape, and size of the craniofacial skeleton in the coronal plane. The most common method is to compare the widths between the right and left jugale points and between the right and left antegonial points [32]. Posteroanterior cephalogram has certain limitations such as landmark identification error, error due to changes in the patient’s head position, magnification, and projection errors [33,34].

Transverse dimensions can also be measured using dental casts, such as dental arch width, which is measured from the cusp tips, and basal arch width, which is measured as the distance between the points at the mucogingival junction adjacent to the respective cusp tips [35]. There is a high chance that dentoalveolar compensations may mask the underlying transverse deficiency while making use of these methods. Diagnostic measurements performed using cephalometric radiographs and dental casts have a questionable accuracy, hence, this study was undertaken to find out the correlation between the transverse basal arches discrepancy and dental compensation with the presence of posterior crossbite by utilizing CBCT width analysis.

CBCT aids in diagnosis by providing evaluation in three planes of space with accuracy, and the additional advantage of minimum radiation [36]. This overcomes the spatial limitation and overlap of molars, which lead to errors in landmark identification on posteroanterior cephalogram. CBCT scans allow evaluators to view coronal cuts in varying depths, which helps in determination of molar and posterior alveolar inclination. It helps in differentiating skeletal and dental transverse problems, thus evaluating the transverse jaw relationships.

Several studies have been conducted where the transverse discrepancy was correlated with different factors. Purva et al. [37] correlated the maxillary transverse dimension with canine impaction, M.K. Alam et al. [38] correlated Bolton’s tooth size ratio with arch width, arch length, and arch perimeter, B.J. Langberg et al. [39] correlated dental and skeletal asymmetry with posterior unilateral crossbite, and Yun-Jin Koo et al. [34] evaluated the maxillo-mandibular transverse measurements to compare the values between normal occlusion and Class III malocclusion groups using dental casts and computed tomography data. R. M. Miner et al. assessed the width of the jaws and the inclination of the first molars in patients with and without crossbites [35,40].

To the best of our knowledge, this study is the first of its kind, which established the correlation between the basal arch width discrepancy and dental compensation due to maxillary deficiency utilizing CBCT analysis. In this study, the transverse basal arch discrepancy was assessed as a modification of Tamburrino et al. [31]. The study sample was divided into a normal maxilla group (discrepancy is 0–5 mm), where the maxillary width is 5 mm wider than the mandibular width as per Andrews [26], and a narrow maxilla group (discrepancy <0 mm). This was in contrast with the study conducted by Miner et al. [35], where the study sample was divided into a crossbite and a non-crossbite group and the skeletal and dental measurements were compared between them. However, our study sample was also divided based on the presence of crossbite “as a secondary comparison” to “no crossbite”, “unilateral crossbite” and “bilateral crossbite” groups.

In the current study, significant differences were observed between the normal maxilla group and narrow maxilla group for maxillary molar compensation, indicating that the amount of compensation seen in the narrow maxilla group was more than that of the normal maxilla group, which was in accordance with a study conducted by Miner et al. [27], where the unilateral crossbite patient group demonstrated obvious dental compensation in the maxillary first molar on the non-crossbite side. Our findings were also in accordance with a study conducted by Yun-Jin Koo et al., where both the maxillary basal arch widths (BAW) measured with CT and BAWs measured with casts were lower in the Class III malocclusion group than in the normal occlusion group, indicating the maxillary transverse dimensions were more in Class III patients compared to patients with normal occlusion [34].

In our study, significant differences were not found between the normal maxilla group and narrow maxilla group for mandibular molar compensation, indicating that the amount of compensation seen in the narrow maxilla group is more or less the same as that seen in the normal maxilla group, which was in accordance with the study conducted by Miner et al. [27], where skeletally, both the bilateral and unilateral crossbite groups had narrower maxillary widths than the controls, but also wider mandibles, with more severe bilateral crossbites, and dentally, the unilateral crossbite group had more upright teeth on the non-crossbite side. These observations were dissimilar to the study conducted by Yun-Jin Koo et al. [34], where the mandibular BAWs measured with CT were significantly narrower in Class I cases compared to cases with a Class III relationship. This could be due to the presence of unilateral or bilateral posterior crossbites in Class III patients due to wider mandibular basal arch widths, which was compensated by maxillary molar inclination.

In this study, the presence of unilateral crossbites and bilateral crossbites was observed more in the narrow maxilla group than in normal maxilla, which may be due to reduced maxillary basal arch widths, thus resulting in a unilateral crossbite. This finding is in parallel with the study conducted by Miner et al. [27], where skeletally, both the bilateral and unilateral crossbite groups had narrower maxillary widths than the control group. Similar findings were obtained by Melink S. et al. [5], where significant correlations between a prolonged pacifier sucking habit and a smaller maxillary arch width and unilateral posterior crossbite have been found.

It is essential to understand the etiology and the nature of the posterior crossbite during case evaluation. The amount of dental compensation in narrow maxilla cases can mask the underlying skeletal discrepancy in the transverse plane [6,15]. Hence, careful evaluation of the teeth angulation is mandatory to achieve excellent treatment outcomes. Recently, the American Board of Orthodontics have adopted the cast-radiograph evaluation system to evaluate orthodontic treatment outcomes [41]. It consists of eight parameters, including the buccolingual inclination of posterior teeth. If an orthodontic treatment’s final result shows buccal-inclined upper posterior teeth, or lingually tipped lower posterior teeth, a mark will be scored, and the evaluation of the treatment outcome will be affected negatively. Hence, skeletal maxillary expansion could help to avoid the need for dental compensation during treatment.

There are different factors that can affect the buccolingual inclination of posterior teeth. For instance, the antero-posterior positioning of upper and lower jaws, or in other words, the horizontal discrepancy, can have an impact on the buccolingual inclination of posterior teeth [6,42,43,44]. Skeletal Class III cases due to a retrognathic maxilla or prognathic mandible usually present with dental compensation of the upper posterior teeth by buccal tipping. This is mainly due to the retrognathic maxilla being small in the transverse plane as well as horizontal plane, or due to the prognathic mandible being positioned more anteriorly [42,43,44]. Moreover, skeletal Class II cases due to a prognathic maxilla or retrognathic mandible might present with a good transverse relationship, but when the lower jaw is positioned in the ideal position—more anteriorly in cases of a retrognathic mandible—by surgical intervention or functional appliances, the transverse discrepancy will be detected [6,43]. Hence, most functional appliances, such as Twin Block or Herbst appliances, come with an expansion component to overcome the underlying transverse discrepancy between the upper and lower jaws [45,46].

Another possible factor that affects the buccolingual inclination of upper or lower posterior teeth is the vertical growth pattern. There are three main types of vertical growth: mesocephalic, brachycephalic, and dolichocephalic. Studies have shown that cases with the short facial type (brachycephalic) usually present with lingually tipped lower posterior teeth, while cases with the long facial type (dolichocephalic) usually present with buccally tipped upper posterior teeth [47,48]. However, the relationship between the buccolingual inclination of posterior teeth is not as strongly affected by the vertical relationship of the upper and lower jaws as by the horizontal relationship [6,42,43,44,47,48].

Results of this study should be interpreted within the context of its methodological strength and limitations. Our findings emphasize the importance of assessing the buccolingual inclinations of molars when making a transverse diagnosis during a clinical examination. Although the sample size in our study satisfied the requirements of the sample size calculation, generalizability of our data is limited as there could be other confounding factors that can affect molar inclinations such as pressure from soft tissue. Through this research, we made an attempt towards the three-dimensional evaluation of the transverse discrepancy, which can be considered as a strength of our study. CBCT is an appropriate method for establishing a transverse diagnosis and ensuring proper skeletal widths of the maxilla and mandible, leading to more accurate treatment planning and better outcomes. However, the following were inherent limitations abiding to our research:

1. We have not made an attempt to assess the dental and skeletal molar relationship.
2. The diagnosis of crossbite in our study was performed using CBCT without any clinical examination.
3. Other confounding factors such as missing teeth, molar uprighting, and growth were not taken into consideration.

Our data only pertain to adult patients, and further studies are needed to establish norms for children of different ages.

## 5. Conclusions

This study has successfully established the correlation between transverse discrepancies and dental compensation, which can in turn lead to unilateral or bilateral posterior crossbite. In our research, we found that maxillary molars were extremely tipped buccally to compensate for the reduced maxillary arch width in the case of a narrow maxilla, and the mandibular molars were largely tipped lingually to compensate for the reduced maxillary basal arch width in order to achieve occlusion with their antagonist teeth.

## Figures and Tables

**Table 1:** Demographic data of the sample.

Group	*n*	Age (y) Mean ± SD	Males/Females	Mx Width Mean ± SD	MB Width Mean ± SD	Difference between Mx and MB Width Mean ± SD
No crossbite	65	31.04 ± 13.23	31/34	55.64 ± 3.48	55.70 ± 3.97	0.06 ± 3.39
Unilateral crossbite	20	33.51 ± 16.85	8/12	55.31 ± 4.07	58.46 ± 4.20	−3.15 ± 3.94
Bilateral crossbite	3	20.33 ± 7.37	1/2	51.63± 3.29	55.88 ± 1.09	−4.25 ± 4.10
Total	88	31.24 ± 13.97	40/48	55.42 ± 3.65	56.34 ± 4.10	−0.91 ± 3.79

Footnote: Mx width—Maxillary width; MB width—Mandibular width.

**Table 2:** Prevalence of posterior crossbite in subjects with normal and narrow maxilla.

Variable	Normal Maxilla	Narrow Maxilla	*p*-Value
No posterior crossbite	38 (90.5%)	27 (58.7%)	0.01 *
Unilateral posterior crossbite	4 (9.5%)	16 (34.8%)	
Bilateral posterior crossbite	0 (0%)	3 (6.5%)	

* Denotes statistical significance.

**Table 3:** Molar axial inclination in subjects with normal and narrow maxilla.

Variable	Normal Maxilla Mean ± SD	Narrow Maxilla Mean ± SD	*p*-Value
Maxillary molars	170.43 ± 10.51°	164.73 ± 10.15°	0.012 *
Mandibular molars	204.54 ± 15.19°	208.64 ± 17.13°	0.25

* Denotes statistical significance.

**Table 4:** Correlation between width difference and molar inclination.

Variable	Pearson’s Correlation Coefficient (r)	*p*-Value
Width difference vs. maxillary molar inclination	−0.37	0.004 *
Width difference vs. mandibular molar inclination	0.117	0.276

* Denotes statistical significance.

## Data Availability

Not applicable.
